# Trends in kidney cancer: exploring the impact of sex and age on stage of disease, and prognosis during the past three decades in Denmark—a DaRenCa study

**DOI:** 10.1007/s10654-025-01236-7

**Published:** 2025-05-14

**Authors:** Johanne Ahrenfeldt, Jesper Jespersen, Jens Ejrnæs Lyngstrand, Laura Iisager, Anna Krarup Keller, Niels Fristrup, Tinne Laurberg, Iben Lyskjær

**Affiliations:** 1 Department of Molecular Medicine, Aarhus University Hospital, Aarhus, Denmark; 2 Steno Diabetes Centre Aarhus, Aarhus University Hospital, Aarhus, Denmark; 3 Department of Urology, Aarhus University Hospital, Aarhus, Denmark; 4 Department of Clinical Medicine, Aarhus University, Aarhus, Denmark; 5 Department of Oncology, Aarhus University Hospital, Aarhus, Denmark

**Keywords:** Disease recurrence, Epidemiology, Kidney cancer, National health care data registries

## Abstract

Renal cell carcinoma (RCC) management has advanced due to increased imaging-based diagnoses and improved therapies for metastatic disease. This nationwide registry-based cohort study examines changes in the number of primary RCC cases, stage at diagnosis, prognosis during the past 30 years in Denmark, and how these are associated with sex and age. All Danish patients aged 18 and older diagnosed with primary RCC from 1992 to 2021 with no prior cancer history (except non-melanoma skin cancer) were included and followed from diagnosis until death or end of follow-up (31-12-2023). Statistical analyses included Pearson's χ2, Cramer's V, Wilcoxon rank-sum test, Kruskal–Wallis, competing risk regression, Cox proportional hazard regression, and Kaplan–Meier. 17,423 RCC patients were identified. Primary RCC cases increased from 2,244 in 1992–1996 to 3,947 in 2017–2021. In this period, the proportion of male patients increased from 59 to 72% (*P* < 0.001). Male patients were younger at diagnosis than female patients (median age 65 vs. 69 years, P < 0.001). Localized cancer cases increased from 44% (N = 983) in 1992–1996 to 70% (N = 2,766) in 2017–2021, while metastatic cases declined from 29% (N = 640) to 17% (N = 652). Median survival for metastatic RCC improved from 4.1 months in 1992–1996 to 13.3 months in 2017–2021. Over three decades, RCC incidence in Denmark has grown, particularly among men, largely due to localized disease, adding pressure on urological departments. The number of metastatic cases remained stable, but survival improved, reflecting advances in early detection and treatment.

**Supplementary Information:**

The online version contains supplementary material available at 10.1007/s10654-025-01236-7.

## Introduction

Kidney cancers account for approximately three percent of all diagnosed cancers, with renal cell carcinoma (RCC) encompassing 90% of diagnosed subtypes [1]. In the past decades, the management of RCC has transformed at multiple stages, including diagnosis and treatment. An increased use of computed tomography (CT) scans has resulted in most cases of RCCs being diagnosed incidentally while investigating other morbidities [2, 3], resulting in a global rise of RCC cases, with uncertainty as to whether this is entirely or only partially explained by the increased use of scans [2]. Additionally, RCC is twice as prevalent in men than in women, potentially due to hormonal and lifestyle differences [4]. As in the rest of the world, previous studies of Danish cohorts have shown increased incidences of RCC, an increased proportion of early-stage tumors, and improved survival for RCC patients [5, 6].

Since 2004, RCC tumors have been classified based on the UICC stage and tumor node metastasis (TNM) classification systems [7]. Tumors of Stage I and II correspond to localized disease with tumors < 7 cm being Stage I, and tumors > 7 cm being Stage II. Stage III tumors are locally advanced tumors. Lastly, Stage IV comprises metastatic tumors that have spread beyond the renal fascia [8]. The degree of surveillance following treatment is for each patient decided based on risk stratification according to a combination of tumor stage and histological parameters (Leibovich score) [9]. Patients not suitable for treatment due to age or other health conditions may be monitored through active surveillance, with additional interventions considered only if the tumor shows signs of progression.

While treatment of localized RCC still largely depends on surgical removal of the tumor, the treatment of metastatic RCC (mRCC) has improved dramatically with the development of targeted therapies in the mid-2000s [10], and then again with the implementation of immune checkpoint inhibitors as first-line treatment in 2018 [11]. The clinical trial for the standard first-line treatment Nivolumab + Ipilimumab showed a median overall survival (OS) of 4 years for mRCC patients [11].

Large retrospective studies with high-quality patient information are needed to further investigate how changes in RCC management have influenced patient outcomes. Danish cohorts are considered highly suitable for population-based studies due to the Danish Civil Registration System (CPR), which enables linkage of national health registers at the individual level and facilitates long-term follow-up (CPR) [12].

This study characterizes all primary incident RCC patients diagnosed in Denmark during the past 30 years, intending to enhance the understanding of how changes in the clinic have impacted the stage at diagnosis and prognosis of RCC patients while investigating the effect of sex and age.

## Methods

### Design and study population

This national cohort study was based on information from the Danish Cancer Registry [13] linked to the Danish National Patient Registry [14], and the Danish Civil Registration System [15]. Here all data are recorded with reference to a civil registration number, a unique personal identification number assigned to all Danish residents. This permits accurate linkage of recorded information at the personal level. [15]

The study population was identified through the Danish Cancer Registry [13], and includes all Danes over 18 years of age diagnosed with primary incident RCC, identified by ICD-10 diagnosis code DC649, from January 1, 1992, to December 31, 2021. Individuals with a prior cancer diagnosis (excluding non-melanoma skin cancer) (Fig. S1) before their primary RCC diagnosis were excluded. Information regarding age at diagnosis, sex, and cancer disease stage was available from the Danish Cancer Registry. Information on migration, date of death, and marital status was added to the dataset from the Danish Civil Registration System. And finally, from the Danish National Patient Registry information regarding hospital admittances and diagnoses was added to the dataset.

### Exposure

To investigate how the stages at diagnosis, recurrence, and survival of RCC patients changed over the years, patients were subdivided into six five-year intervals, based on their year of diagnosis: 1992–1996, 1997–2001, 2002–2006, 2007–2011, 2012–2016, 2017–2021.

### Outcome

For patients diagnosed before 2004, disease stage is defined as ‘localized cancer’, ‘regional spread’, and ‘metastatic cancer’ in the Danish Cancer Registry. After 2004, the cancer stage was registered and classified by the TNM stage [16]. To make the periods comparable, tumors diagnosed after 2004 were categorized as: Localized when the tumor was coded any T stage, N0 and M0, Regional spread when the tumor was coded any T stage, N1-N3 and M0, and Metastatic if the tumor was coded any stage T, any stage N and M1. Finally, RCC tumors diagnosed after 2004 were categorized according to the UICC stage classification [7].

Recurrence was defined as patients receiving a diagnosis of either distant or regional metastases (DC76*-DC80*, DC991*, DC649X, and DC649M) with a parent diagnosis of kidney cancer (DC649) in the Danish National Patient Registry [14].

Overall survival (OS) was defined as the duration from diagnosis to the time of death (event), or until the most recent available update on the patients, i.e. migration date (censoring event) or until December 31, 2023, for the patients who were still alive. The patients diagnosed in 2021 were omitted from the three-year survival analyses, as three-year follow-up was not available for these patients.

### Covariates

Pre-existing comorbidity at the time of RCC diagnosis was defined with the Charlson Comorbidity Index (CCI) [17]. We used the Danish National Patient Register [14] to obtain inpatient visit information related to the CCI. The score was categorized into CCI 0, CCI 1–2, CCI > 2.

Marital status was obtained from the Danish Civil Registration System [15], and categorized as married (including civil partnerships), widowed, divorced, or unmarried (including annulled marriages or no registered partnerships).

### Statistical methods

Descriptive data was presented as the median with interquartile ranges (Q1, Q3) for continuous variables and proportions (n, %) for categorical variables. The number of RCC cases was calculated for the six five-year intervals (year of diagnosis), and characterized by age, sex, and stage of the tumor (both by disease stage, UICC stage, and T stage).

Pearson's χ2 test was utilized to assess the dependence between categorical variables, and Cramer's V was employed to evaluate the effect size for this dependence, with a Cramer's V ≥ 0.1 considered indicative of a weak association. The Wilcoxon rank-sum test was employed to examine significant differences between two groups of continuous variables. To determine significant differences between three or more independent groups, the Kruskal–Wallis test was utilized.

Recurrence and all-cause mortality were analyzed using the Fine-Gray subdistribution hazard model for competing risk regression (accounting for death as a competing event) and Cox proportional hazard regression, respectively. The main exposure was the six five-year intervals, with 1992–1996 as the reference, and adjustments were made stepwise for age and sex (Model 1), marital status (Model 2), and comorbidity (Model 3). To illustrate the change in overall survival over time, three-year survival analyses were conducted using Kaplan–Meier curves for death after an RCC diagnosis in one of the six five-year intervals. All analyses were performed separately within each disease stage and each UICC stage.

To investigate whether an aging population drives the increasing number of patients, we analyzed the demographic characteristics of the Danish population. We obtained the demographic population data from Statistics Denmark [18] from 1992 to 2021 and calculated crude incidence rates, sex specific incidence rates and age-standardized incidence rates. The latter using the European standard population as the reference [19].

Data analysis was performed in R version 4.3.3 [20] with the packages; tidyverse (RRID: SCR_019186), gtsummary (RRID:SCR_021319), survminer (RRID:SCR_021094), survival (RRID: SCR_021137), tidycmprsk (RRID:SCR_025053) and vcd [21].

P-values below 0.05 were considered statistically significant.

## Results

### Patient population

Between 1992 and 2021, 21,473 individuals in Denmark were diagnosed with primary RCC. After excluding 4,050 patients (19%), 17,423 individuals (81%) were included in the final analysis (Fig. S1). As indicated in Table 1, the number of primary RCC diagnoses has continuously increased during the past 30 years and has nearly doubled from 448 patients annually in 1992–1996 to 789 cases annually in 2017–2021. When accounting for changes in the age distribution of the population, the age-standardized incidence rate still shows a notable increase, from 14 cases per 100,000 Danes in 1992 to 18 cases per 100,000 in 2021 (Fig. S2a).

**Table 1:** Baseline characteristics of the study cohort separated into five-year intervals

	N (not Unknown)	1992–1996	1997–2001	2002–2006		2007–2011		2012–2016		2017–2021	
		N = 2,244^a^	N = 2,287^a^	N = 2,434^a^		N = 2,998^a^		N = 3,513^a^		N = 3,947^a^	
**Age**	17.423	68 (58, 75)	68 (57, 76)	66 (57, 75)		65 (57, 74)		66 (57, 73)		67 (57, 74)	
**Sex**	17.423										
Female		925 (41%)	903 (39%)	861 (35%)		1,002 (33%)		1,179 (34%)		1,107 (28%)	
Male		1,319 (59%)	1,384 (61%)	1,573 (65%)		1,996 (67%)		2,334 (66%)		2,840 (72%)	
**Localized**	14.175										
Localized cancer		983 (44%)	1,032 (45%)	1,068 (44%)		1,226 (41%)		1,958 (56%)		2,766 (70%)	
Regional spread		287 (13%)	251 (11%)	119 (4.9%)		64 (2.1%)		69 (2.0%)		75 (1.9%)	
Metastatic		640 (29%)	698 (31%)	788 (32%)		832 (28%)		667 (19%)		652 (17%)	
Unknown		334 (15%)	306 (13%)	459 (19%)		876 (29%)		819 (23%)		454 (12%)	
**UICC stage**	9.311				**2004–2006**						
Stage I		0	0	344 (14%)	344 (22%)	748 (25%)		1,277 (36%)		1,907 (48%)	
Stage II		0	0	176 (7.2%)	176 (11%)	223 (7.4%)		238 (6.8%)		259 (6.6%)	
Stage III		0	0	124 (5.1%)	124 (8%)	232 (7.7%)		439 (12%)		610 (15%)	
Stage IV		0	0	524 (22%)	524 (34%)	847 (28%)		683 (19%)		680 (17%)	
Unknown		2,244 (100%)	2,287 (100%)	1,266 (52%)	397 (25%)	948 (32%)		876 (25%)		491 (12%)	
**T stage**	10.113				**T stage sum**		**T stage sum**		**T stage sum**		**T stage sum**
**T1**		0	0	347 (14%)	**519 (21%)**	484 (16%)	**1,110 (37%)**	220 (6.3%)	**1,670 (48%)**	157 (4.0%)	**2,191 (56%)**
T1a		0	0	64 (2.6%)		350 (12%)		920 (26%)		1,466 (37%)	
T1b		0	0	108 (4.4%)		276 (9.2%)		530 (15%)		568 (14%)	
**T2**		0	0	355 (15%)	**355 (15%)**	481 (16%)	**481 (16%)**	440 (13%)	**440 (13%)**	417 (11%)	**417 (11%)**
**T3**		0	0	165 (6.8%)	**228 (9.4%)**	266 (8.9%)	**485 (16%)**	127 (3.6%)	**712 (20%)**	82 (2.1%)	**899 (23%)**
T3a		0	0	53 (2.2%)		197 (6.6%)		568 (16%)		784 (20%)	
T3b		0	0	0 (0%)		0 (0%)		0 (0%)		14 (0.4%)	
T3c		0	0	10 (0.4%)		22 (0.7%)		17 (0.5%)		19 (0.5%)	
**T4**		0	0	144 (5.9%)	**144 (5.9%)**	239 (8.0%)	**239 (8.0%)**	124 (3.5%)	**124 (3.5%)**	99 (2.5%)	**99 (2.5%)**
Unknown		2,244 (100%)	2,287 (100%)	1,188 (49%)		683 (23%)		567 (16%)		341 (8.6%)	

The pathological T stage is reported in substages when possible, with each of the four main stages summarized in bold

^a^Median (Q1, Q3); n (%)

Median age at diagnosis was relatively consistent at 65–68 years (Table 1). However, men were diagnosed four years younger than women (median 65 vs. 69, Table S1). Among men, age at diagnosis rose within each UICC stage for Stages I, II, and IV over time (Table S2), while no significant changes were observed for female patients (Table S2).

### Stage migration

**Fig. 1:**
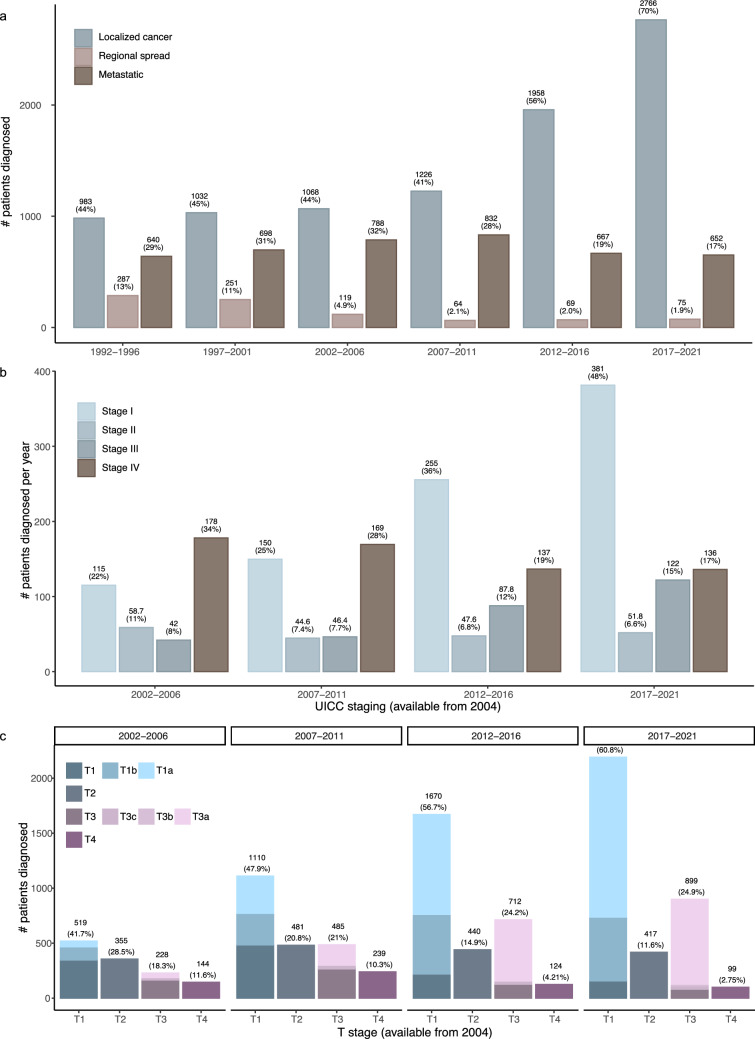
Distribution of cancer stage and at diagnosis over time. **a** Number of cases categorized as Localized, Regional spread, or Metastatic cancer over 30 years, presented in five-year intervals. **b** Number of cases per year at each tumor stage since 2004, when UICC staging was implemented, in five-year intervals. **c** Number of cases within each T stage since 2004. The bars are colored by the detailed T stages. Above the bars, the number of patients is shown. The percentages are of the total number of cases, diagnosed in the given time interval, except for (**c**) where it is of the out of the cases with T stage information

From 1992 to 2021, the proportion of localized RCC cases increased from 44 to 70%, while primary metastatic cases declined from 29 to 17% (Table 1 and Fig. 1a, *P* < 2.2 × 10^–16^, Cramer’s V = 0.204). Despite this shift, the absolute number of metastatic cases remained relatively stable at 137–190 patients annually (Table 1 and Fig. 1a). Cases with regional spread decreased drastically from 13% in 1992–1996 to 1.9% in 2017–2021, although the most drastic decline occurred in the five years 2002–2006, likely due to changes in registration (Table 1 and Fig. 1a), thus we will not further investigate this group.

Focusing on the UICC stage at diagnosis from 2004 to 2021, a shift in stages is also observed here (*P* < 2.2 × 10^–16^, Cramer’s V = 0.15). Stage I cases increased markedly from 22% in 2002–2006 to 48% in 2017–2021, whereas a decrease was observed for Stage II and IV, most pronounced for Stage IV (from 34% in 2002–2006 to 17% in 2017–2021) (Table 1 and Fig. 1b).

Over time, the proportion of male patients increased from 59% in 1992–1996 to 72% in 2017–2021 (*P* < 0.001) (Table 1). Correspondingly, the incidence rate among males rose markedly, from 14 cases per 100,000 males in 1992 to 28 cases per 100,000 in 2021, whereas the incidence rate among females remained stable at approximately 10 cases per 100,000 females (Fig. S2b). While the stage distribution/proportion of regional and metastatic tumors was similar between sexes over time (Tables S1, S2, and S4).

The excluded patients with previous cancers follow the study cohort trends, except that they are older. When examining all 21,473 individuals the results differ by only 0–3 percentage points (data not shown).

### Prognosis

Patient survival has significantly improved in the 30-year period. For localized and metastatic RCC, hazard ratios (HR) for overall survival have decreased steadily across five-year intervals in both unadjusted and adjusted models (Table 2), particularly for UICC Stages III and IV (Table S5).

**Table 2:** Overall survival of primary RCC after the year of diagnosis, unadjusted and adjusted analysis for each disease stage

	Unadjusted		Model 1^a^		Model 2^b^		Model 3^c^	
	HR [95% CI]	p-value	HR [95% CI]	p-value	HR [95% CI]	p-value	HR [95% CI]	p-value
*Localized cancer*								
1992–1996	Ref							
1997–2001	0.91 [0.83;1.00]	0.05	0.88 [0.80;0.96]	0.0066	0.87 [0.79;0.95]	0.0036	0.85 [0.77;0.93]	< 0.001
2002–2006	0.74 [0.67;0.82]	< 0.001	0.71 [0.64;0.79]	< 0.001	0.71 [0.64;0.78]	< 0.001	0.68 [0.61;0.75]	< 0.001
2007–2011	0.57 [0.51;0.63]	< 0.001	0.53 [0.47;0.58]	< 0.001	0.52 [0.47;0.57]	< 0.001	0.49 [0.44;0.55]	< 0.001
2012–2016	0.46 [0.42;0.51]	< 0.001	0.42 [0.38;0.47]	< 0.001	0.40 [0.36;0.45]	< 0.001	0.37 [0.34;0.42]	< 0.001
2017–2021	0.36 [0.32;0.41]	< 0.001	0.31 [0.28;0.35]	< 0.001	0.30 [0.27;0.34]	< 0.001	0.27 [0.24;0.31]	< 0.001
*Metastatic*								
1992–1996	Ref							
1997–2001	0.85 [0.77;0.95]	0.0041	0.85 [0.76;0.95]	0.0036	0.84 [0.76;0.94]	0.0021	0.84 [0.76;0.94]	0.0022
2002–2006	0.73 [0.66;0.81]	< 0.001	0.74 [0.67;0.83]	< 0.001	0.74 [0.66;0.82]	< 0.001	0.73 [0.66;0.81]	< 0.001
2007–2011	0.60 [0.54;0.67]	< 0.001	0.60 [0.54;0.66]	< 0.001	0.59 [0.53;0.66]	< 0.001	0.58 [0.52;0.65]	< 0.001
2012–2016	0.50 [0.45;0.56]	< 0.001	0.50 [0.45;0.56]	< 0.001	0.49 [0.44;0.55]	< 0.001	0.48 [0.43;0.54]	< 0.001
2017–2021	0.42 [0.38;0.48]	< 0.001	0.40 [0.36;0.45]	< 0.001	0.39 [0.35;0.44]	< 0.001	0.39 [0.34;0.44]	< 0.001

Cox proportional hazard regression for overall survival

Risk of death after RCC diagnosis based on year of diagnosis within five year intervals

^*a*^Model 1: adjusted for age at diagnosis and sex

^*b*^Model 2: adjusted like Model 1 and further adjusted for marital status

^*c*^Model 3: adjusted like Model 2 and further adjusted for CCI

**Fig. 2:**
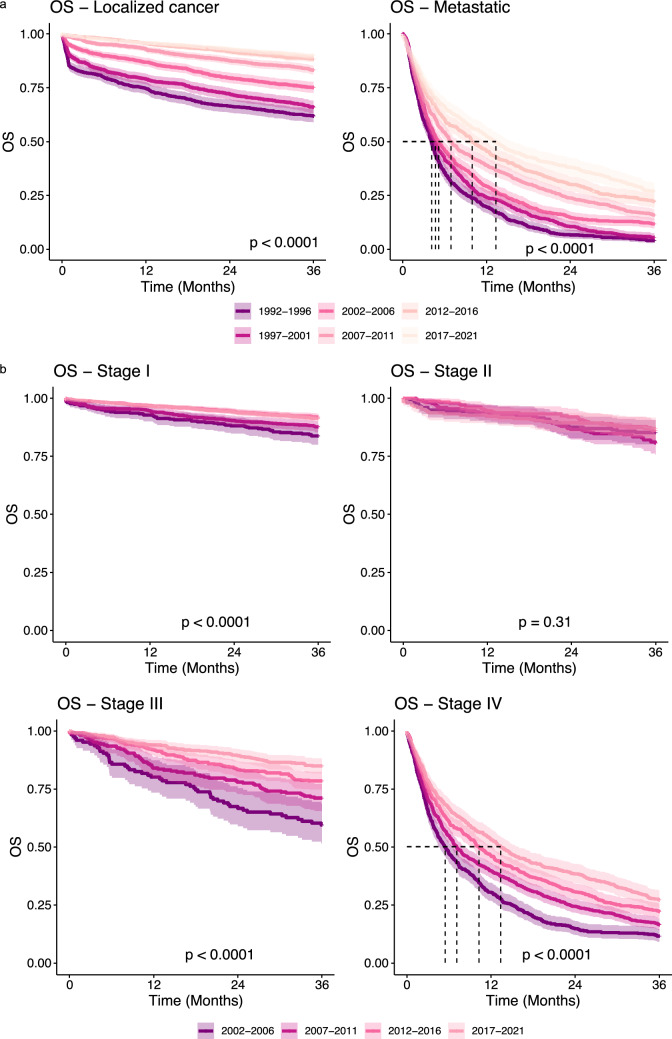
Survival over time. **a** Kaplan Meier curves of the overall survival (OS) are stratified by five-year intervals with an independent plot for each disease stage. **b** Kaplan Meier curves of the overall survival (OS) are stratified by five-year intervals with an independent plot for each UICC stage. Median survival is illustrated by dashed lines, if a 50% death rate is reached within 3 years

The improvements in overall survival across almost all stages are illustrated in Fig. 2. For metastatic RCC, median survival increased from 4.1 months in 1992–1996 to 13.3 months in 2017–2021—a 324% increase. Cumulative survival rates increased across all stages (Tables S6, S7). For localized RCC, one- and five-year survival rates increased from 75 and 52% in 1992–1996 to 96% and 82% in 2017–2021. For metastatic RCC, survival rates rose from 20% and 2.4% to 54% and 17% in the same period (Table S6). Focusing on UICC Stage III cases from 2002–2021, survival rates at one, and five years improved from 80 and 48% in 2002–2006 to 94% and 73% in 2017–2021 (Table S7), highlighting substantial progress in RCC survival over time.

The risk of recurrence for localized RCC cases has decreased, and in 2017–2021 the HR was 0.64 compared to 1992–1996 (Table 3). For all stages, except Stage III, the risk of recurrence decreased over time (Table S8).

**Table 3:** Competing risk regression analysis for recurrence after the year of primary RCC diagnosis, unadjusted and adjusted analysis for localized cancer

	Unadjusted		Model 1^a^		Model 2^b^		Model 3^c^	
	HR [95% CI]	p-value	HR [95% CI]	p-value	HR [95% CI]	p-value	HR [95% CI]	p-value
Localized cancer								
1992–1996	Ref							
1997–2001	1.19 [1.01;1.40]	0.040	1.17 [1.00;1.38]	0.055	1.17 [0.99;1.38]	0.061	1.18 [1.00;1.40]	0.044
2002–2006	1.17 [0.99;1.38]	0.060	1.15 [0.97;1.36]	0.10	1.14 [0.97;1.35]	0.11	1.16 [0.99;1.37]	0.071
2007–2011	0.94 [0.79;1.11]	0.5	0.92 [0.78;1.09]	0.3	0.91 [0.77;1.08]	0.3	0.93 [0.78;1.10]	0.4
2012–2016	0.86 [0.73;1.00]	0.057	0.84 [0.72;0.99]	0.033	0.83 [0.71;0.98]	0.026	0.86 [0.74;1.01]	0.073
2017–2021	0.64 [0.54;0.76]	< 0.001	0.63 [0.53;0.74]	< 0.001	0.62 [0.52;0.73]	< 0.001	0.64 [0.54;0.76]	< 0.001

Competing Risk Regression for recurrence

Risk of recurrence (event) after RCC diagnosis based on year of diagnosis within five year intervals, with death as competing risk

^a^Model 1: adjusted for age at diagnosis and sex

^b^Model 2: adjusted like Model 1 and further adjusted for marital status

^c^Model 3: adjusted like Model 2 and further adjusted for CCI

## Discussion

This large population-based study highlights a 76% rise in primary RCC cases in Denmark during the 30-year period (1992–2021), driven largely by an increase in Stage I at diagnosis, particularly in male patients. The number of metastatic cases has remained stable, while survival has increased, highlighting the impact of early detection and treatment advancements over time.

During the observation period, the Danish population aged 60 years and older increased by 46% [18], partly explaining the observed rise in primary RCC incidence; however, the increase in age-standardized incidence from 14 to 18 cases per 100,000 indicates that factors beyond demographic shifts are contributing significantly to this upward trend (Fig. S2a). As the main increase was seen for Stage I cancers, this likely reflects increased incidental detecting through expanded CT scan use, which increased from 230,000 scans in 2003 to 990,000 scans in 2017 in Denmark [22]. This aligns with previous findings suggesting that advanced and more frequent imaging detects small tumors, which would otherwise have been detected at a more severe stage of disease or potentially not at all, increasing the number of diagnosed RCC patients [23].

Over the past 30 years, the mean age at diagnosis has increased for men but not for women. This aligns with the 7.1-year rise in male life expectancy in Denmark from 1992 to 2021 [24], though the 5.5-year increase for women [24], did not translate to higher age at diagnosis. This may be explained by the earlier detection of smaller tumors in women, offsetting the gains in general health. Additionally, sharper declines in smoking rates among men [25] and sex differences in diagnostic practices or incidental findings may help explain this pattern.

The proportion of male RCC patients rose from 59% in 1992–1996 to 72% in 2017–2021, a trend not observed globally [26, 27]. This is unlikely due to diagnostic bias as it spans all stages. This finding was unexpected, as dietary and smoking habits in Denmark have become increasingly similar between men and women [25]. The original predominance of male RCC patients has been suggested to stem from the high levels of estrogen receptors present in RCC tumors, which may suppress tumor development in women [28]. This is supported by data linking late menarche (> 15 years) and hysterectomy to increased RCC risk [29, 30]. While oral contraceptives have been connected to multiple adverse effects after their broad introduction in the late 1960s [31], most depend on a systemic increase of estrogen levels [32].This elevation in estrogen has been hypothesized to exert a protective effect against RCC development [33]. A large meta-analysis found that women using oral contraceptives were 11% less likely to develop RCC, with an increased effect with prolonged use [33]. The high use of oral contraceptives in Denmark compared to the rest of the world could explain why this effect is more pronounced in this study [34, 35]. While we are unable to explore the biological meaning of these results directly, the reduced proportion of women being diagnosed with RCC in Denmark highlights the importance of investigating the hormonal regulation of RCC development.

We found that the survival rates for RCC have improved steadily over the 30 years analyzed, with the metastatic patients’ median survival increasing from 4.1 months in 1992–1996 to 13.3 months in 2017–2021. This effect may largely be attributed to the introduction of new treatments, with immune checkpoint inhibitors showing drastic improvement in survival compared to patients treated with interleukin-2 [36–38] which was the standard of care in Denmark until the mid-2000s [39]. Early detection may also help by extending the treatment window. In 2007, Denmark implemented an initiative to increase tumor diagnostic scans and hasten treatment, potentially making the effect of early intervention especially pronounced in Denmark [40].

As this study is based solely on the Danish population, it cannot be directly compared to other populations with different healthcare systems, demographic characteristics, and cancer screening practices. In addition, the uncertainty linked to register-based studies allows for potential inaccuracies in the recorded data [41], such as the patient's marital status or underreported comorbidities, which could potentially impact the results of this study. Although our models for survival and recurrence account for important covariates, such as age at diagnosis, sex, marital status, CCI, and stage, we acknowledge that other potential confounders—including race/ethnicity, socioeconomic status, geographic region, healthcare access, and histopathological subtype—could influence the observed associations. The absence of these variables represents a limitation of this study.

Taking these caveats into account, the trends observed in this study indicate that the pattern identified in Denmark is consistent with global patterns and may serve as a useful point of reference for other countries, particularly those where detailed clinical parameters and demographic information for each patient are unobtainable.

## Conclusions

This study provides the first comprehensive analysis of Danish primary RCC patients over 30 years, supporting a global trend of increased early-stage diagnoses and improved survival, particularly among metastatic cases. We found a previously unreported rise in the proportion of male patients across all disease stages, which is crucial to investigate further. While this research is limited to the Danish population, it serves as a reference for understanding the evolution of primary RCC patient demographics in the context of increased use of imaging techniques and advancements in treatments.

## Supplementary Information

Below is the link to the electronic supplementary material.

Supplementary file1 (PDF 100 KB)

Supplementary file2 (XLSX 28 KB)
