# Supplementary material for: Trends in kidney cancer: exploring the impact of sex and age on stage of disease, and prognosis during the past three decades in Denmark—a DaRenCa study
Source: Eur J Epidemiol. 2025 May 14;40(5):527–36. doi: 10.1007/s10654-025-01236-7 (PMC12170715; doi:10.1007/s10654-025-01236-7)
Supplement: Supplementary file 1 — Supplementary file1 (PDF 100 KB) [file 10654_2025_1236_MOESM1_ESM.pdf]

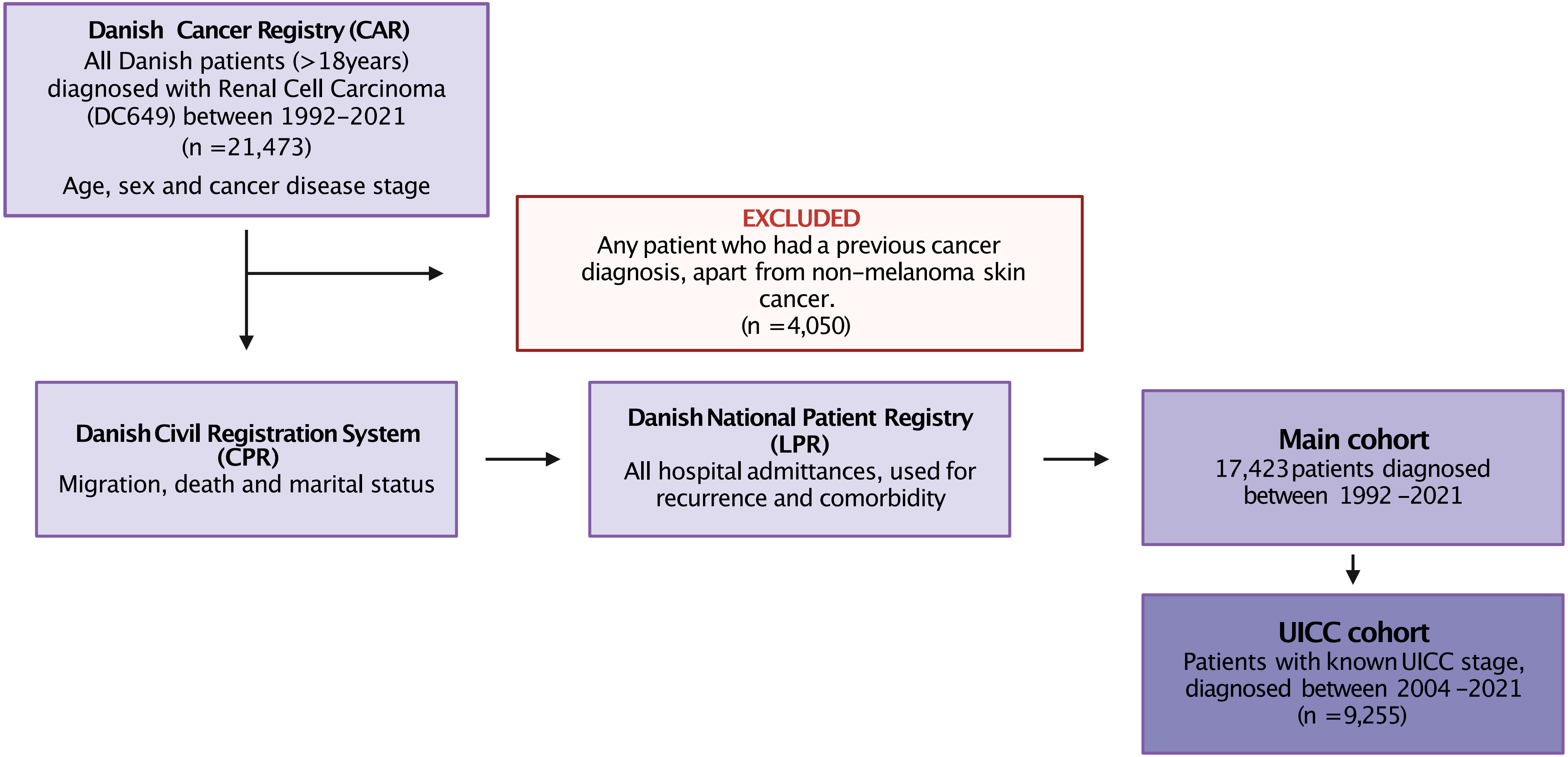

Figure S1. Study design. This flowchart shows the study design and exclusion criteria for this study and the number of patients removed and remaining in the final cohorts.

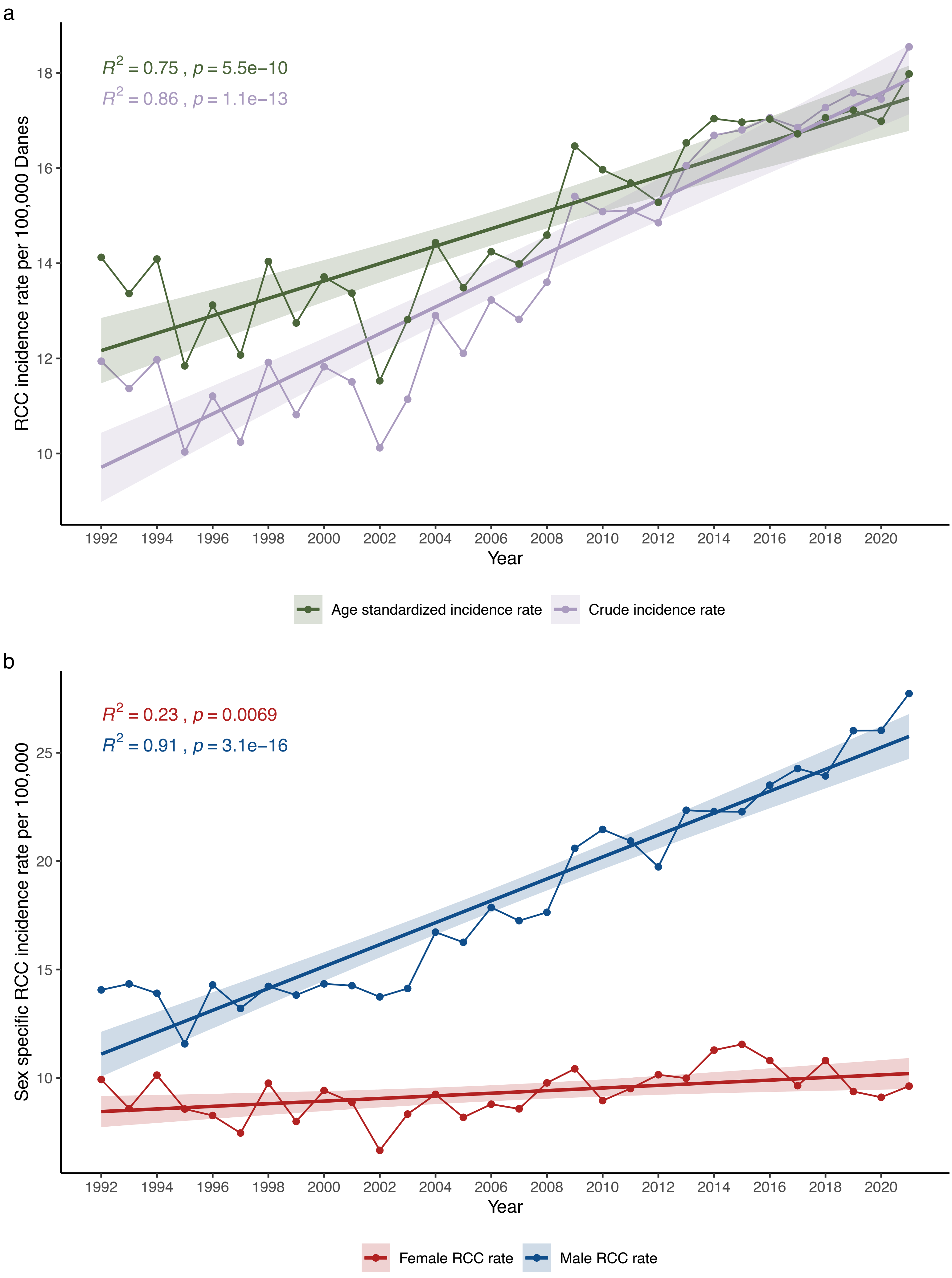

Figure S2. Incidence rates. a) Age standardized incidence rate over the 30 years shown together with the raw non−standardized incidence rate. b) Sex specific incidence rates per 100,000 male or female Danish citizens over the 30 years.
